# Functional genomics of *Plasmodium falciparum* using metabolic modelling and analysis

**DOI:** 10.1093/bfgp/elt017

**Published:** 2013-06-22

**Authors:** Stepan Tymoshenko, Rebecca D. Oppenheim, Dominique Soldati-Favre, Vassily Hatzimanikatis

**Keywords:** Plasmodium falciparum, central carbon metabolism, systems biology, flux-balance analysis, constraint-based modelling, in silico gene essentiality

## Abstract

*Plasmodium falciparum* is an obligate intracellular parasite and the leading cause of severe malaria responsible for tremendous morbidity and mortality particularly in sub-Saharan Africa. Successful completion of the *P. falciparum* genome sequencing project in 2002 provided a comprehensive foundation for functional genomic studies on this pathogen in the following decade. Over this period, a large spectrum of experimental approaches has been deployed to improve and expand the scope of functionally annotated genes. Meanwhile, rapidly evolving methods of systems biology have also begun to contribute to a more global understanding of various aspects of the biology and pathogenesis of malaria. Herein we provide an overview on metabolic modelling, which has the capability to integrate information from functional genomics studies in *P. falciparum* and guide future malaria research efforts towards the identification of novel candidate drug targets.

## INTRODUCTION

According to the recent ‘World Malaria Report’ by the World Health Organization, malaria remains a major healthcare issue, being responsible for more than 200 million cases and hundreds of thousands of deaths in 2010 alone [1]. An efficient and cost-effective artemisinin-based treatment is now available, but the emergence of resistance in malaria parasites, as a result of drug treatments, urges the development of medicines with new targets and novel mechanisms of action. Despite tremendous research efforts and the passing of a decade since the publication of the *Plasmodium falciparum* genome sequence [2], about a half of the genes still remain annotated as coding for ‘hypothetical proteins’ or ‘conserved hypothetical proteins’ [3]. These genes, and especially those restricted to *Plasmodium* or apicomplexan species, are of particular interest as their unique nucleotide sequences may provide higher selectivity for new antiparasitic drugs. However, in the absence of tools for high-throughput gene knockdown such as RNAi [4], the identification and validation of the essentiality of these genes for the parasite remains a major bottleneck [3]. Available experimental approaches for establishing gene essentiality in *P. falciparum* are cumbersome due to several unique properties of the pathogen. First, it possesses an extremely AT-rich genome along with an unusually low frequency of homologous recombination, which makes it refractory to genetic manipulation such as gene replacement [3]. Second, *in vitro* cultivation is a delicate process [5] that is still mainly restricted to the intraerythrocytic stages. Third, the limitation of using primates as an animal model makes the *in vivo* assessment of gene essentiality very limited and expensive. Nevertheless, emerging experimental breakthroughs hold promises for cost-effective gene knockdown strategies of the *P. falciparum* essential genes at a high-throughput scale [6, 7]. In this context, there is a need to list genes of immediate interest, which should be validated as antimalarial targets once affordable experimental means are available.

Metabolic modelling is a modern approach of systems biology that, among several other applications, has been extensively exerted to predict gene essentiality in various bacteria including a number of pathogenic species [8]. Computational (i.e. *in silico*) metabolic models offer a cost-effective pipeline to identify putatively indispensable metabolic functions that, in the case of pathogens, represent potential targets for medical intervention [9]. Eight years after the publication of the first computational model of a tentative metabolic network of *P. falciparum*, it has become evident that reconstruction and analysis of *in silico* models is a valuable tool for studying various aspects of the pathogen [10–14]. In this study, we aim to review and provide an outlook on the current state and contribution of *in silico* metabolic modelling efforts to functional genomics studies on the deadly malaria parasite.

## THE PATH FROM METABOLIC MAPS TO ‘CONTEXT FOR CONTENT’ MODELS

With the constantly decreasing cost of high-throughput measurements, the tendency to generate very large -omics datasets is emerging and this holds also true for *P. falciparum* [15]. There are two distinct approaches to high-throughput measurements: hypothesis-driven studies generate large datasets to prove or falsify a hitherto existing hypothesis, while hypothesis-free studies primarily rely on a thorough analysis of datasets without presumptions aiming at the formulation of conclusions and testable hypotheses. Importantly, hypothesis-free approaches require an appropriate context, i.e. a framework of related prior knowledge, within which an obtained dataset can be interpreted. In the case of functional genomic studies, *in silico* metabolic models have been shown to provide such context, thus enabling researchers to use available datasets (i.e. content) to improve and challenge the models and to derive additional, non-intuitive insights. In particular, this need for an explicit, up-to-date context is a driving force for the progress from a scope of generic biochemical knowledge to organism-specific (or life stage specific) metabolic networks, which we will discuss further.

In the past two decades, several databases of biochemical reactions and metabolic pathways have been developed to provide a systematic and comprehensive overview of metabolism. A pertinent example is the Kyoto Encyclopedia of Genes and Genomes (KEGG) Pathway Database [16], which has established itself as an encyclopedia of biochemical knowledge and the first point of reference for numerous academic and industrial researches. However, KEGG and similar large databases are more universal than organism-specific, thus they do not cover some crucial aspects of the *P. falciparum* metabolism, such as cofactor utilization, compartmentalization and proteins involved in the transport of metabolites. The web-resource Malaria Parasite Metabolic Pathways (MPMP) is essentially the product of an extensive manual revision of KEGG maps according to known and recently established metabolic features of the *P. falciparum* intraerythrocytic life stage. Furthermore, integration with other web-resources facilitates quick access to additional information and related primary literature [17]. MPMP is probably the most comprehensive and up-to-date knowledge database on *P. falciparum’s* metabolism, but unfortunately the lack of any application-programming interface limits the access of a broad research community to this high-quality data. Utility of MPMP for research purposes has been demonstrated via interpretation of the expression patterns for the genes involved in the pentose-phosphate pathway using the corresponding maps [18]. This led the authors to postulate that the oxidative part of the pathway is predominantly active during the early stage of the intraerythrocytic replication cycle, whereas the non-oxidative part is activated only during the later stages [18]. In contrast to this approach, further attempts to comprehend metabolic fluxes in the malaria parasite aimed at going beyond the study of a particular pathway and ultimately led to the reconstruction of large-scale models with hundreds of metabolites and interconnected reactions involved [10–14].

Graph-based models (GBMs) were built to comprehend and analyse the metabolic capabilities of *P. falciparum* in a systematic manner. GBMs represent metabolism as graphs with nodes (to denote metabolites) and links between them (respective metabolic reactions) [19]. The links only represent the possibility of interconversion for a particular metabolite into another one (justified by the correspondingly annotated genes), regardless of all the properties of the reaction including its stoichiometry. A natural advantage of GBMs is that they require minimal input information and can assess well-annotated parts of a given metabolic network while skipping unclear ones. However, such representation of a metabolic network without capturing its mass-balance property is not a suitable framework for incorporation of experimental information, thus making the scope of GBM-based methods in metabolic modelling rather limited. Nowadays, graph-based metabolic reconstructions and topological analysis are largely obsolete and have been replaced by more comprehensive constraint-based approaches.

Over the past decade flux-balance analysis (FBA) has been established as a leading approach for studying constraint-based models (CBMs) of cellular metabolism [20]. Constraint-based metabolic models for FBA are based on two-dimensional arrays, where each row represents a metabolite and each column corresponds to a reaction, which, as a rule, is linked to a certain enzyme and gene in the organism [21]. Each intersection of row and column contains a numerical value standing for the stoichiometric coefficient of a given metabolite in a given reaction. Such notation enables the explicit and quantitative description of a metabolic network and allows the imposing of the first basic constraint—mass balance. A model that only accounts for mass balance is largely undefined and further constraints are imposed to enrich the range of feasible solutions with biologically realistic ones. The scope of additional constraints is constantly growing and currently includes thermodynamic (tFBA), regulatory (rFBA) and other constraints inherent in cellular metabolism (reviewed in [22]) and constraints inferred from experimental data.

CBMs, unlike GBMs, do require a pre-defined objective towards which utilization of available substrates should be optimized [23]. A common objective function for fast-growing cells is the biomass reaction (other plausible objectives are reviewed in [24]). It represents cellular replication as a reaction that consumes pre-defined amount of metabolites referred to as precursors of biomass. Existence of a solution implies that the stoichiometric array describes at least one uninterrupted pathway that leads to the transformation of externally supplied substrates into biomass precursor for each of the precursors. To satisfy this requirement, the model-building process often involves the inclusion of ‘orphan reactions’, for which no enzyme-coding gene has been annotated yet [25].

A conventional reconstruction workflow [25] clearly defines the list of enzymes that should be found in the genome to complete the pathways and meet experimentally observed metabolic behaviour. With this approach, putative metabolic functions have been proposed for 17 genes of *Leishmania major*, which previously had no functional annotations [26]. Although these assignments, often based on moderate sequence identity, are not sufficient proofs of the suggested functions, they do represent a set of testable hypotheses for experimental validation. Such a list of orphan reactions provides an invaluable guidance for functional genomics studies into *P. falciparum* that has more than 2000 genes without even putative functional annotation.

One of the most important applications for CBMs of pathogenic species is the capability to predict potential vulnerabilities in their metabolism [9]. Often metabolic networks are redundant and contain more than one chain of reactions to produce certain biomass precursors. To explore this redundancy, FBA enables an attempt at the simulation of growth when each of the reactions in the model is removed in a one-at-a-time manner. Whenever production of biomass is blocked without a certain reaction, the latter is classified as essential. In a similar manner, FBA can simulate outcomes of withdrawal of enzymes or genes by attempting to simulate growth when utilization of all the reactions associated with an enzyme or gene is disabled. We refer the reader to a comprehensive publication on *in silico* essentiality studies in the CBM of *Saccharomyces cerevisiae* [27] for further details and examples.

## OUTLOOK OF *IN SILICO* METABOLIC MODELS FOR *P. FALCIPARUM*

Several studies to date have focused on the comprehensive reconstruction of the metabolic network of *P. falciparum* (Table 1). Early studies produced several GBMs [11, 14, 28] that considered the set of metabolic activities reported at the time without taking into account information on the compartments they occur in nor the differences in life-stage-specific metabolism of the pathogen. As mentioned before, without consistency in mass-balance relationships, these models were not suitable for integration of experimental data and allowed only qualitative predictions of gene essentiality. Nevertheless, during the reconstruction process putative functions were suggested for hundreds of genes previously annotated as coding for a ‘hypothetical protein’ [14]. These first modelling efforts gave a broad overview of the metabolic capabilities of *P. falciparum* annotated in its genome when compared with the expected ones and provided a solid foundation for building the modern, more comprehensive reconstructions discussed hereafter.

**Table 1:** Comparison of *in silico* metabolic reconstructions of *P. falciparum*

Authors (year of publication)	Information about the model^a^			
	Metabolites	Reactions	Genes	Compartments^b^
Yeh *et al.* (2004) [14]	525	696	—	—
Fatumo *et al.* (2009) [11]	554	575	—	—
Huthmacher *et al.* (2010) [12]	**P**: 1622 **E**: 566	**P**: 1375 **E**: 437	**P**: 579	**P**: c, m, a, n, r, v, g; **E**: e, c
Plata *et al.* (2010) [13]	915	1001	366	P: e, c, m, a
Bazzani *et al.* (2012) [10]	**P**: 1622 **H**: 1149	**P**: 1394 **H**: 2539	**P**: 579 **H**: 704	**P**: c, m, a, n, r, v, g; **H**: c, r, g, l, m, n, p, b, s

The first two models [11, 14] were built using graph-based approach and the following are constraint-based models [10, 12, 13].

^a^‘P’ denotes the model of the parasite, ‘E’ human erythrocyte, ‘H’ human hepatocyte.

^b^Abbreviated names of compartments: e, extracellular space; c, cytosol; m, mitochondrion, a, apicoplast; n, nucleus; v, digestive vacuole; r, endoplasmic reticulum; g, Golgi complex; l, lysosome; p, peroxisome; b, bile canaliculus; s, sinusoidal space.

Using a constraint-based approach, Huthmacher *et al.* [12] assembled the first compartmentalized, mass-balanced and life-stage-specific model of *P. falciparum* metabolism. Through *in silico* simulations, the authors identified enzymatic activities that are essential for proliferation of the parasite. Thirty of the *in silico* essential reactions were catalyzed by enzymes with no homologues in the human proteome (*E*-value > 0.075). These were ranked as targets of particular interest based on evidence of activity during the multiple stages of *P. falciparum* life cycle studied and/or the presence in SuperTarget database [29] as candidates for treatment of other infections [12]. Furthermore, the natural environment of the parasite was simulated by embedding its CBM into the metabolic reconstruction of a human erythrocyte. This limited the substrate accessibility to only those available in the host cell milieu. Such constraints at the cellular interface and constraints on reaction fluxes, deduced from gene expression profiles (obtained on different life stages), not only allowed the model to retrieve known directions of metabolite exchanges between the host cell and the parasite, but also identified several inconsistencies with experimental data, which need further investigation [12].

The second CBM developed independently by Plata *et al*. [13] also took account of compartmentalization of the intracellular space and mass-balance constraints. The results of gene deletions performed *in silico* were found to be in correspondence with reports in the primary literature: 100% agreement when compared with gene deletion studies and 70% in case of enzymatic inhibition experiments. Comparison of the number of essential genes in the models of *P. falciparum* and *S. cerevisiae* confirmed the notion that the parasite likely possesses significantly lower metabolic flexibility to bypass single-gene deletions when compared with free-living organisms with similar genome sizes [13]. Forty genes were suggested as potential drug targets due to their *in silico* essentiality and extremely low or absent sequence identity to human proteins [13]. The essentiality of one of these genes encoding the nicotinate mononucleotide adenylyltransferase was verified using an experimental inhibitor, which caused an arrest of *P. falciparum* proliferation at an IC_50_ of 50 µM in *in vitro* culture [13]. In addition, Plata *et al.* [13] were the first to report *in silico* double-gene deletion simulations in *P. falciparum* leading to the identification of 16 pairs of genes that were predicted as non-essential by single-gene knockout simulation but resulted in a dramatic impairment of the metabolism if targeted simultaneously.

Holzhutter and co-workers have developed the most recent CBM of the *P. falciparum* metabolic network [10] by updating their previous PlasmoNet1 model [12]. In PlasmoNet2 [10] they have added new transport reactions based on metabolomics data [30] and removed one reaction according to an updated version of KEGG database. Through integration of PlasmoNet2 with the CBM of human hepatocyte [31], they evaluated *in silico* the essentiality of *P. falciparum* genes in liver stage and assessed, using a ‘reduced fitness’ approach, effects of targeting enzymes that are homologous and predicted to be essential both in the host and the pathogen [10].

Comparison of the essentiality predictions made by the aforementioned models is not a trivial task. First, because the study by Plata *et al*. aimed at predicting the essentiality of genes, whereas the other studies assessed the essentiality of enzymes, the predictions made do not overlap when gene-to-enzyme relations are not one-to-one. Second, a gene or enzyme may be absent from the list of ‘predicted as essential’ not only because it has been predicted as non-essential, but possibly, also due to the fact that the corresponding metabolic process simply is not included in the model of interest. Third, essentiality predictions in CBMs are directly dependent on the set of metabolites included into their biomass reaction, so that differences in assumed biomass composition directly affect the results of *in silico* simulations. For an overview of *P. falciparum* genes and enzymes predicted as essential in existing models, we refer the reader to the Supplementary Table 1. The table also provides the reader with literature references on the experimental assessment of genes/enzymes predicted to be essential by the different models.

An important simplification common to all the aforementioned models is the *ad hoc* assignment of directionality to the reactions, which are pre-set either as all reversible [12] or assumed to have the same reversibility as in the metabolic models of non-related organisms [13]. In principle, the directionality of a reaction is subject to its thermodynamic properties and spurious *ad hoc* assignments might violate this fundamental constraint. This issue has been addressed rigorously in genome-scale metabolic networks of several organisms [32–34] by implementation of thermodynamic constraints on reaction directionality as an extension to conventional FBA methodology.

Overall, metabolic modelling of *P. falciparum* to date lags several years behind the similar efforts for the model eukaryote *S. cerevisiae*; there exist a few independently reconstructed models that often lack consistency with each other due to the differences in the reconstruction workflows, the sources of primary information, the level of complexity and the varying degree of comprehensiveness. Similarly to the trends in modelling of the yeast metabolism, we expect the emergence of reconciliation efforts that will aim at obtaining a consensus, up-to-date CBM of *P. falciparum.* Recently developed workflows for manual [35] and semi-automated [36] reconciliations of existing metabolic models may significantly facilitate these efforts. There are several other areas in which we foresee room for upcoming improvements: the first, as mentioned before, is a systematic implementation of thermodynamic constraints in the models of *P. falciparum*; the second is a deliberate revision of the objective function (i.e. biomass reaction) to make it consistent with the actual biomass composition of the parasite at the life stage of interest; the third is an experimental verification and quantification of the uptake fluxes present in the models. These improvements are likely to make *in silico* predictions of gene essentiality more reliable and also expand the number of metabolic functions currently known to be essential for *P. falciparum*.

Metabolic reconstruction efforts to date have summarized the results of decades of experimental research on the metabolism of *P. falciparum* in the form of *in silico* models, which not only reproduce the prior knowledge, but also provide novel insights*.* Nevertheless, the fact that cultivation of the parasite in a fully defined medium is still impossible clearly highlights that some important metabolic peculiarities remain to be discovered. The promising direction is the utilization of the CBMs as frameworks of current knowledge, which can then be challenged, refined and constrained by various high-throughput datasets as discussed in the following section.

## HIGH-THROUGHPUT DATA AND METABOLIC MODELS: ‘CONTENT FOR CONTEXT’

As mentioned above, CBMs hold a great potential to incorporate various types of experimental data as content for the context of computational metabolic networks. In this section of the review we provide an outlook of the currently published studies that have integrated computational modelling and experimental research efforts for each type of high-throughput data introduced in Table 2.

**Table 2:** Overview of the high-throughput methods applied for functional genomics of *P. falciparum* and available options for integration of the data with CBMs

Approach	Examples of the methods	Maximal coverage	Integration with CBMs
Genomics	BLASTP [2], metaSHARK [37], DETECT [38]	ca. 50%	Used as input data for building CBMs [25]; validation of *in silico* gene essentiality [39]
Transcriptomics	DNA microarray [40–42], RNA-seq [43]	ca. 99%	Used to constrain CBMs [12, 13]; reviewed in [44]
Proteomics	2D LC-MS/MS [45], nano-LC-MS/MS [46]	ca. 45%	Used to constrain CBMs [47]
Metabolomics	HPLC-MS/MS [30, 48], ^1^H-NMR [49]	ca. 15-20%	Used to constrain CBMs [32, 34], reviewed in [33]
Fluxomics	^13^C-NMR [50, 51], ^14^C-NMR [52]	Less than 1%	Used to constrain CBMs, reviewed in [53, 54]

### Genomics

Genomics studies of *P. falciparum* have yielded so far several complete genome sequences (for 3D7 and IT strains [55]); however, their functional annotation remains far from being complete [3]. Nonetheless, these partially annotated genomes gave rise to numerous genome-wide transcriptomics and large-scale proteomics studies as well as made possible the reconstruction of the parasite’s metabolic network *in silico*.

High-throughput functional genomics of *P. falciparum* is a nascent field in malaria research since only a limited number of studies have succeeded in generating single-gene mutant parasite clones at a large scale. The largest coverage reported to date was achieved with a forward genetics approach based on transposon mutagenesis using the transposable element *piggyBac* [56]. The collection regroups about 200 mutant parasite lines that cover only a modest part of the *P. falciparum* genome. Among those, 24 single-gene disruptions caused severe growth defects *in vitro*, which might lead to lethal phenotypes *in vivo* [56]. A major drawback of this method is its inability to reveal essential genes due to the haploid state of the malaria parasite throughout most of its life stages. Although specific design of the transposon with an integrated inducible promoter has been proposed to assess essential genes [6], it still remains to be proved practicable. In contrast, *in vitro* essentiality data of the genetically tractable parasite *Trypanosoma brucei* have been obtained for nearly all the coding sequences of its genome using the RNA-interference target sequencing approach [57].

The value of the gene essentiality datasets is reinforced by the possibility of *in silico* simulation of similar genetic perturbations*.* This offers reciprocal benefits for experimental and computational research: CBMs can often suggest the underlying reason for viable/non-viable phenotypes of knockout strains and, otherwise, reveal the weak points, where the model should be improved. Accordingly, the algorithm GrowMatch [39] has been applied for comparison of the high-throughput datasets of viable/non-viable single- and double-gene knockout mutants in *S. cerevisiae* with *in silico* gene essentiality predictions [27]. This approach has led to over a hundred corrections in the model (e.g. inclusion of additional reactions, compounds and genes as well as changes in biomass reaction), each supported by literature evidence and largely improved consistency of the model with the existing experimental data [27]. Once a successful high-throughput forward or reverse genetic technique is established for *P. falciparum,* GrowMatch or a similar algorithm could validate the computational model, to improve understanding of the obtained results and produce hypotheses for experimental investigation, applicable to malaria drug research.

### Transcriptomics

Transcriptomic profiling appears to be the one of most common high-throughput methods applied in malaria research; numerous gene expression datasets are available in PlasmoDB database (http://www.plasmodb.org) for different lineages of the parasite under various conditions. Although the scope of gene expression data is growing, new increasingly advanced algorithms to integrate these datasets into CBMs are also being developed [44]. To date, two models of *P. falciparum* metabolism have incorporated available transcriptomics data as constraints [12, 13] to represent life-stage-specific metabolic features of the parasite.

Huthmacher *et al*. integrated their model with several life-stage-specific gene expression profiles [40–42, 58, 59] to avoid, whenever possible, utilization of reactions that are likely to be inactive based on the abundance of the corresponding messenger ribonucleic acids (mRNAs) [12]. This approach has allowed the authors to infer plausible metabolite exchanges between the human erythrocyte and the parasite, as well as to predict directionality of the pathways that cannot be easily inferred from gene expression data alone. The predictions of the host–parasite metabolite exchanges were in significantly better agreement with the physiology reported in literature when fluxes were allowed through any reaction for which the mRNA was absent at the life-stage-specific transcriptome but had been present earlier (up to 12 preceding hours) [12]. This observation is consistent with the earlier study that found a significant time delay between maximal gene expression and peak of accumulation of the corresponding proteins [60].

Plata *et al.* [13] also attempted to integrate gene expression data [30, 61] into their model to predict the shifts in the extracellular (intraerythrocytic) abundance of some metabolites between ring to trophozoite and trophozoite to schizont stages. The authors assumed that a higher influx of a metabolite into the parasite will lower the abundance of the substrate molecule in the cytosol of the infected erythrocyte, whereas a higher efflux rate of a metabolite from the parasite will increase its intraerythrocytic abundance. The computational predictions were verified against the existing metabolomics data [30] and had an average accuracy of 70%.

With major improvements in accuracy and sensitivity thresholds that can be achieved by modern RNA-seq approaches [43], utility of transcriptomic studies will increase significantly for experimental and *in silico* functional genomics.

### Proteomics

The first two large-scale proteomics datasets published in 2002 [45, 46] provided a relatively high coverage (ca. 45% and 23% respectively) of the expected proteome of *P. falciparum* [62]. While delivering only semi-quantitative results, these studies provided the unique possibility to verify and correct the genome annotation in terms of assignments of open reading frames, splicing patterns and to confirm the presence of particular enzymes in different stages of infection.

Several approaches have been used to generate quantitative proteomics datasets. Nirmalan *et al.* [63] have established a method for fully quantitative proteomics using [^13^C_6_^15^N_1_]-labelled isoleucine to recognize *de novo* synthesized proteins. Following this method, Prieto *et al*. [64] quantified 1253 proteins in *P. falciparum* trophozoites before and after exposure of the parasite to chloroquine and artemisinin, allowing identification of proteins involved into the parasite’s response to treatment with these conventional antimalarial drugs. A recent alternative method for quantitative proteomics relies on externally supplied, known amounts of proteins of interest, obtained using QconCAT technique [65]. QconCAT-derived proteins are labelled with heavy isotopes and serve both as markers for identification of the similar unlabeled proteins and a scale for their quantification [65]. This approach offers a new level of sensitivity and holds promise for comprehensive high-resolution quantitative proteomics in *P. falciparum*.

Taking into account the complexity of the interplay between mRNA and protein abundances (thoroughly examined in [66]), the studies that combine both transcriptomic and proteomic measurements are of particular interest. As a result of such an integrated approach applied to *P. falciparum*, time-delayed correlation between peaks of transcription and maximal abundance of the proteins has been observed for all the glycolytic enzymes with the exception of enolase [60]. However, for several enzymes, accumulation of the mRNA did not correlate with the changes in the abundance of the corresponding enzymes over a complete intraerythrocytic replication cycle [60]. Mair *et al.* [67] demonstrated that in the *Plasmodium* species some genes can be transcribed but not expressed due to translational repressions.

Despite the availability of the data and necessary computational methods, proteomics have not been used systematically in the development and analysis of CBMs of *P. falciparum*. Meanwhile however, it has been done for an intercellular pathogen *Trypanosoma cruzi*: Roberts *et al*. have integrated in their CBM a proteomics dataset that constrains fluxes through reactions whenever the corresponding enzyme is not detected in life-stage-specific proteome*.* Using this constraint the authors aimed at making the model the most representative possible of the metabolic state of the pathogen at a particular stage of its life cycle. Initially this resulted into an over-constrained model that was unable to simulate growth, suggesting that even though enzymes for some reactions were not detected in the proteome they were likely to be present. On the other hand, constraints inferred from the proteomics data corrected those reaction essentiality predictions that were not in agreement with experimental data without these constraints [47].

Although a complex interplay between concentrations of mRNAs and enzymes with the fluxes through the corresponding reactions remains to be elucidated, utilization of transcriptomics and proteomics information together can ensure higher confidence that an enzyme of interest is (or is not) present in the life stage of interest. For instance, it is reasonable to assume that a reaction for which both enzyme and transcripts cannot be detected is most likely not to occur and should be tightly constrained in the stage-specific CBMs.

### Metabolomics

Several recent reviews discuss in detail the methodologies and techniques that are currently used for metabolomics of various organisms [68–70] and malaria parasites in particular [71]. The largest metabolomics profile to date was obtained by liquid chromatography coupled to a tandem mass-spectrometry (LC-MS/MS) analysis of both uninfected and *P. falciparum*-infected human erythrocytes [30]. Relative changes in the concentrations of about 90 metabolites were monitored in both the medium and cell lysates within a whole replication cycle, with measurements every 8 h [30]. A further attempt to correlate these results with gene expression data obtained at the same time points of the infection revealed that despite a common periodic pattern in gene expression, only less than a third of the measured metabolite abundances fluctuated periodically [30]. The coverage of the *P. falciparum* putative metabolome achieved in this study is between 15% and 20% (estimated similarly to [72]) and compares with the metabolomics of *Leishmania donovani* promastigotes [72]*.* Currently, no algorithm is available to directly incorporate the relative concentration values in CBMs. Even so, Plata *et al*. used this data as a reference to verify their *in silico* predictions as discussed above. Furthermore, the dataset [30] gives a valuable insight into actively consumed and secreted metabolites, an aspect of the metabolic fate that can be used as a constraint by CBMs.

Quantification of absolute concentration values using MS is technically possible, although it is hampered by the need for a standard solution for each metabolite [48, 73]. On the contrary, nuclear magnetic resonance (NMR) techniques do not require external standards neither for identification nor for quantification of metabolites as the integral of the output signal is proportional to the concentration of the studied nuclei. Despite its lower sensitivity compared with the modern MS methods [70], ^13^C-NMR can identify metabolites that are otherwise undetectable by MS due to their low ionization potential (e.g. glycerol as reported in [74]). Using ^1^H-NMR, the concentrations of more than 50 metabolites were measured in cell extracts of *P. falciparum* trophozoites [49]. The unbiased nature of the method also enabled identification of some unexpected metabolites (e.g. aminobutyric acid and buffering agent HEPES) present in relatively high concentrations in lysates of the parasite cells [49].

An important drawback of the current metabolomics studies of *P. falciparum* is that, for metabolites present in more than one compartment (e.g. cytosolic and mitochondrial adenosine diphosphate), the measured concentration only reflects an average value, which may differ significantly from the actual concentration in each of the compartments. Metabolomics on separate organelles is an emerging field in functional genomics research, as exemplified in algea [75], which will enrich and verify current knowledge on the subcellular localization of various metabolic processes. The issue of compartmentalization is especially complex in the case of malaria parasites. Indeed, these obligate intracellular parasites develop in either hepatocytes or erythrocytes in the intermediate host. Moreover, the parasite harbours two symbiotic organelles: the mitochondrion and the apicoplast (relic of a plastid organelle acquired by engulfment of an algae), both of which are host metabolic pathways that are crucial to the central carbon metabolism of *P. falciparum*.

### Fluxomics

Fluxomics is a largely unexplored area in malaria research, while for experimentally amenable species it represents a relatively well-established and rapidly developing field [53, 54]. Incorporation of the measured values of flux through the reactions present in CBMs can significantly improve the accuracy of the models by reducing the uncertainty in the ranges and distributions of metabolic fluxes. To the best of our knowledge, the only fluxomics studies in *P. falciparum*, to date, are the assessments of influx rates for single substrates: glucose (in infected and non-infected human erythrocytes [50, 51]), isoleucine [76], pantothenate [52] and inorganic phosphate [77]. Due to the indispensability of these substrates for the parasite, incorporation of these flux values may represent overriding constraints for the CBMs.

In the case of model organisms, e.g. *S. cerevisiae*, measured metabolic fluxes were included into the CBMs and allowed *in silico* resolution of experimentally observed metabolic features that could not be inferred otherwise—neither from transcriptomics nor proteomics data [54]. A relevant example is a large increase in glycolytic flux, which can be maintained by the yeast exposed at low levels of oxygen without changing the expression levels for involved genes [78]. This led to an important conclusion that fluxes in primary metabolism are more likely to be controlled via regulation of enzymatic activities and not by changes in gene expression as in secondary metabolism [54].

Overall, out of numerous methods developed for the integration of experimental data with CBMs, only a modest proportion have hitherto been applied to pathogenic organisms and *P. falciparum* in particular. We argue that this is not only due to the complexity of the experimental study of pathogens, but also due to the fact that a majority of the *in silico* methods discussed above were initially designed for free-living organisms. Although the methods are, in principle, applicable to intracellular pathogens, some aspects unique to the parasites may be crucial for obtaining relevant computational results. Examples of such aspects would be the common absence of a clearly defined set of substrates and by-products for the metabolism of intracellular parasites, the changes in composition of biomass across multiple stages of their life cycles or sub-optimal utilization of available substrates, etc. These issues and the aforementioned experimental challenges represent the area where we expect forthcoming improvements, leading to a better understanding of metabolic peculiarities encoded in the genome of *P. falciparum*.

## FUTURE DIRECTIONS

Obligate intracellular parasitism of *P. falciparum* represents a significant challenge for medical treatment, as it is virtually impossible to avoid impact of antimalarial drugs on the host. Thus, high selectivity is an essential prerequisite for all potential drug candidates. However, many of the genes and enzymes predicted as being essential in *P. falciparum* do possess a variable level of sequence identity to their human counterparts [10, 12, 13], suggesting that an interference with them may also affect uninfected host cells. To assess this phenomenon *in silico*, the ‘reduced fitness’ approach was applied by Bazzani *et al*. [10] to the CBM of *P. falciparum* embedded into the model of a human hepatocyte. This method can estimate quantitatively how production of biomass would be affected in the host and the parasite’s metabolic networks when the flux through a targeted reaction is gradually reduced to the same extent in both models. Although it shows a purely theoretical sensitivity of the host and the parasite to inhibition of a certain enzymatic activity, this approach should be considered with caution as it neglects some important aspects, which cannot be captured by CBMs.

FBA is indeed suitable for simulation of the outcomes for gene knockout experiments, when targeted enzymatic activity is abolished completely. However, a realistic simulation of the effects of reduced enzymatic activity (i.e. action of a drug) requires careful consideration of kinetic and thermodynamics properties. Ultimately, even if one assumes identical kinetic properties for inhibited enzymes in the infected host cell and its parasite, sensitivity of the flux through the catalyzed reaction to the same amount of inhibitor can be different simply if the expression level of this enzyme is not the same in these organisms [79].

Metabolic control analysis (MCA) considers all the relevant properties to provide a more realistic estimation about the extent to which flux through a reaction of interest is sensitive to the action of an inhibitor [80–82]. Using the methodologies from MCA, one can also predict whether the inhibition of a target enzyme will have a selective effect on the parasite relative to its host. Using kinetic modelling and MCA, Bakker *et al.* [83] have demonstrated that glyceraldehyde-3-phosphate dehydrogenase and phosphoglycerate kinase may represent promising targets for the treatment of sleeping sickness due to drastically different susceptibility of glycolytic fluxes to inhibition of these enzymes in human and in African trypanosome.

A major limiting factor for application of MCA to metabolic models of *P. falciparum* is the need to integrate the kinetic parameters of enzymes in the model and the concentration profile of key metabolites [84]. To date, such experimental information exists only for a modest part of the CBMs of a relatively small number of organisms [85], which is of limited applicability [86, 87]. The recently developed ORACLE framework can be used to perform MCA under significant uncertainty to provide a guidance and a quantitative ranking for drug target identification [84]. Kinetic modelling and MCA represent the next promising field of research for a fine-grained evaluation of the pathways that contain *in silico* predicted drug targets. These efforts may also provide a valuable guidance for further development in the functional genomics of *P. falciparum*, whereas analytical technologies will provide the information necessary for building a kinetic model of its metabolic network.

## Supplementary Material

Supplementary Data

## SUPPLEMENTARY DATA

Key Points

- Modelling efforts in *P. falciparum* focused on revealing indispensable metabolic functions have expanded the list of putatively essential genes and enzymes.
- Interpretation of high-throughput data in the context of CBMs yields new, non-intuitive insights about functions of metabolic genes and offers testable hypotheses for experimental research.
- Kinetic modelling approaches will enable the integration of a broad range of data from functional genomics, such as metabolomics, and address the limitations of the CBMs.
- Reliable kinetic modes of metabolic networks will accelerate drug target identification and rational drug development.
