# Supplementary material for: Functional genomics of Plasmodium falciparum using metabolic modelling and analysis
Source: Brief Funct Genomics. 2013 Jun 22;12(4):316–27. doi: 10.1093/bfgp/elt017 (PMC3743259; doi:10.1093/bfgp/elt017)
Supplement: Supplementary Data [file supp_12_4_316__index.html]

Functional genomics of Plasmodium falciparum using metabolic modelling and analysis — Functional genomics of Plasmodium falciparum using metabolic modelling and analysis — Supplementary Data 

# Functional genomics of *Plasmodium falciparum* using metabolic modelling and analysis

## Supplementary Data

files

**Files in this Data Supplement:**

- Supplementary Data - docx file
