# Supplementary material for: Functional genomics of Plasmodium falciparum using metabolic modelling and analysis
Source: Brief Funct Genomics. 2013 Jun 22;12(4):316–27. doi: 10.1093/bfgp/elt017 (PMC3743259; doi:10.1093/bfgp/elt017)
Supplement: Supplementary Data [file supp_elt017_Supplementary_Table_1.docx]

| **Supplementary Table 1**. Single gene/enzyme deletions predicted to cause impairment of metabolic network of *P. falciparum*.  **Abbreviations:** GR: Growth Reducing, SGR: Slight Growth Reducing, "+": predicted as essential, "−": predicted as non-essential or not present in the model; " \| ": delimiter for genes that code isozymes, "&": delimiter for genes that code subunits of multienzyme complex, "ORPHAN": no gene encoding this enzyme has been identified yet. | | | | | | | | | |
| --- | --- | --- | --- | --- | --- | --- | --- | --- | --- |
| **Gene ID (old/new)** | **Enzyme** | **E.C. number** | **Yeh et al.** | **Fatumo et al.** | **Huthma-cher et al.** | **Plata et al.** | **Bazzani et al.** | **Experimental anlaysis based on drugs or genetic approaches** | **Influence** |
| PF11_0059/PF3D7_1104800 | Pantothenate transporter | − | **−** | **−** | **−** | **+** | **−** | ([Saliba et al., 1998](#_ENREF_79)) | Drug |
| PF11_0169/PF3D7_1116200.1 | Pyridoxine/pyridoxal 5-phosphate biosynthesis enzyme | − | **−** | **−** | **−** | **+** | **−** | No evidence |  |
| PFE0410w/PF3D7_0508300 | Dihydroxyacetone phosphate transporter (apicoplast) | − | **−** | **−** | **−** | **+** | **−** | ([Banerjee et al., 2012b](#_ENREF_6)) | Genetic  (Essential) |
| PFE1510c/PF3D7_0530200 | Phosphoenolpyruvate transporter (apicoplast) | − | **−** | **−** | **−** | **+** | **−** | ([Banerjee et al., 2012b](#_ENREF_6)) non essential | Genetic  (non- essential) |
| PFF0450c/PF3D7_0609100 | Iron transporter | − | **−** | **−** | **−** | **+** | **−** | No evidence |  |
| MAL13P1.206/PF3D7_1340900 | Phosphate transporter (cytoplasm) | − | **−** | **−** | **−** | **SGR** | **−** | ([Saliba et al., 2006](#_ENREF_80)) |  |
| PFI1020c/PF3D7_0920800 | IMP dehydrogenase | 1.1.1.205 | **−** | **−** | **−** | **−** | **+** | ([Webster and Whaun, 1982](#_ENREF_105)) | Drug |
| ORPHAN | Shikimate dehydrogenase | 1.1.1.25 | **−** | **−** | **−** | **+** | **−** | No evidence |  |
| PF14_0641/PF3D7_1467300 | 1-deoxy-D-xylulose 5-phosphate reductoisomerase | 1.1.1.267 | **−** | **−** | **+** | **+** | **−** | ([Cassera et al., 2007](#_ENREF_19); [Jomaa et al., 1999](#_ENREF_41); [Odom and Van Voorhis, 2010](#_ENREF_65)) | Drug - Genetic |
| PF13_0141/PF3D7_1324900 \| PF13_0144 (new annotated PF3D7_1325200 oxidoreductase) | lactate dehydrogenase | 1.1.1.27 | **−** | **−** | **−** | **+** | **+** | ([Razakantoanina et al., 2000](#_ENREF_75)) ([Vivas et al., 2005](#_ENREF_100)) | Drug  (Highly toxic drug) |
| PF14_0520/PF3D7_1454700 | 6-phosphogluconate dehydrogenase (decarboxylating) | 1.1.1.44 | **−** | **−** | **−** | **SGR** | **−** | No evidence |  |
| PF14_0373/PF3D7_1439400 & PF10_0120/PF3D7_1012300 & PF14_0248/PF3D7_1426900 | mitochondrial ubiquinol-cytochrome C reductase | 1.10.2.2 | **−** | **−** | **+** | **−** | **+** | ([Biagini et al., 2012](#_ENREF_9)) | Drug |
| ORPHAN | 2-octaprenylphenol hydroxylase | 1.14.13.- | **−** | **−** | **+** | **+** | **−** | No evidence |  |
| PFF1130c/PF3D7_0623500 \| PF08_0071/PF3D7_0814900 | superoxide dismutase | 1.15.1.1 | **−** | **−** | **+** | **−** | **+** | ([Soulere et al., 2003](#_ENREF_87)) | Drug |
| PFA0225w/PF3D7_0104400 | 4-hydroxy-3-methylbut-2-enyl diphosphate reductase | 1.17.1.2 | **−** | **−** | **−** | **+** | **−** | ([Vinayak and Sharma, 2007](#_ENREF_99)) |  |
| PF14_0352/PF3D7_1437200 & PF10_0154/PF3D7_1015800 \| PF14_0053/PF3D7_1405600 | ribonucleotide reductase | 1.17.4.1 | **+** | **+** | **+** | **+** | **+** | ([Chakrabarti et al., 1993](#_ENREF_20); [Munro and Silva, 2012](#_ENREF_60)) | Drug |
| PF10_0221/PF3D7_1022800 | (E)-4-hydroxy-3-methylbut-2-enyl-diphosphate synthase | 1.17.7.1 | **−** | **−** | **−** | **+** | **−** | No evidence |  |
| PF14_0598/PF3D7_1462800 | Glyceraldehyde-3-phosphate dehydrogenase (phosphorylating) | 1.2.1.12 | **−** | **−** | **−** | **+** | **−** | No evidence |  |
| PF13_0070/PF3D7_1312600 \| PFE0225w/PF3D7_0504600 | 3-Methyl-2-oxobutanoate dehydrogenase (BCKDH) | 1.2.4.4 | **−** | **+** | **−** | **−** | **−** | No evidence |  |
| PFF0730c/PF3D7_0615100 | enoyl-ACP-reductase | 1.3.1.9 | **+** | **−** | **+** | **−** | **−** | ([Vaughan et al., 2009](#_ENREF_98); [Yu et al., 2008](#_ENREF_108)) | Genetic |
| PF11_0436/PF3D7_1142400 | Coproporphyrinogen oxidase | 1.3.3.3 | **−** | **−** | **+** | **+** | **−** | No evidence |  |
| PF10_0275/PF3D7_1028100 | Protoporphyrinogen oxidase | 1.3.3.4 | **−** | **−** | **−** | **+** | **−** | ([Nagaraj et al., 2010](#_ENREF_61); [Ramya et al., 2007](#_ENREF_73)) | Drug |
| PFF0160c/PF3D7_0603300 | Dihydroorotate oxidase (fumarate)/dihydroorotate dehydrogenase | 1.3.3.1/1.3.98.1 | **−** | **−** | **+** | **+** | **−** | ([Baldwin et al., 2005](#_ENREF_4); [Deng et al., 2009](#_ENREF_27); [Gujjar et al., 2011](#_ENREF_35); [Painter et al., 2007](#_ENREF_67)) | Drug |
| PFD0830w/PF3D7_0417200 | dihydrofolate reductase | 1.5.1.3 | **−** | **+** | **−** | **+** | **+** | ([Jiang et al., 2000](#_ENREF_40); [Massimine et al., 2006](#_ENREF_54)) | Drug |
| PFF1490w/PF3D7_0630700 | Methylenetetrahydrofolate dehydrogenase (NADP+), methenyltetrahydrofolate cyclohydrolase | 1.5.1.5, 3.5.4.9 | **−** | **−** | **−** | **+** | **−** | ([Massimine et al., 2006](#_ENREF_54)). | Drug |
| PFI0735c/PF3D7_0915000 | NADH dehydrogenase | 1.6.99.3 | **+** | **+** | **−** | **−** | **+** | ([Biagini et al., 2006](#_ENREF_10); [Uyemura et al., 2004](#_ENREF_96)) | Drug |
| PF14_0192/PF3D7_1419800.1 | glutathione reductase | 1.8.1.7 | **−** | **−** | **+** | **−** | **+** | ([Biot et al., 2004](#_ENREF_11); [Grellier et al., 2010](#_ENREF_34); [Luersen et al., 2000](#_ENREF_53); [Zhang et al., 1988](#_ENREF_110)) | Drug |
| PFI1170c/PF3D7_0923800.1 | thioredoxin reductase | 1.8.1.9 | **−** | **−** | **+** | **+** | **+** | ([Krnajski et al., 2002](#_ENREF_45)) | Genetic |
| multiple genes | cytochrome C oxidase | 1.9.3.1 | **−** | **−** | **−** | **+** | **+** | No evidence |  |
| PFL1780w/PF3D7_1236800 | Protein S-isoprenylcysteine-O-methyltransferase | 2.1.1.100 | **−** | **−** | **+** | **−** | **−** | No evidence |  |
| MAL13P1.214/PF3D7_1343000 | phosphoethanolamine N-methyltransferase | 2.1.1.103 | **−** | **−** | **−** | **−** | **+** | ([Witola et al., 2008](#_ENREF_106)) | Genetic |
| PFD0830w/PF3D7_0417200 | thymidylate synthase | 2.1.1.45 | **+** | **+** | **+** | **+** | **+** | ([Jiang et al., 2000](#_ENREF_40); [Nduati et al., 2005](#_ENREF_62)) | Drug |
| MAL7P1.130/PF3D7_0724300 \| PFI0815c/PF3D7_0916600 | 3-Demethylubiquinone-9,3-O-methyltransferase | 2.1.1.64 | **−** | **+** | **+** | **−** | **−** | No evidence |  |
| PF14_0534/PF3D7_1456100 \| PFL1720w/PF3D7_1235600 | serine hydroxymethyltransferase | 2.1.2.1 | **−** | **−** | **−** | **−** | **+** | No evidence |  |
| MAL13P1.221/PF3D7_1344800 | aspartate carbamoyltransferase | 2.1.3.2 | **−** | **−** | **+** | **+** | **+** | ([Banerjee et al., 2012a](#_ENREF_5)) | Drug |
| PFF0530w/PF3D7_0610800 | Transketolase | 2.2.1.1 | **−** | **−** | **−** | **+** | **−** | No evidence |  |
| MAL13P1.186/PF3D7_1337200 | 1-deoxy-D-xylulose-5-phosphate synthase | 2.2.1.7 | **−** | **−** | **−** | **+** | **−** | ([Cassera et al., 2004](#_ENREF_17); [Cassera et al., 2007](#_ENREF_19)) | Drug |
| PFL0620c/PF3D7_1212500 \| PF13_0100/PF3D7_1318200 | Glycerol 3-phosphate O-Acyltransferase | 2.3.1.15 | **−** | **+** | **−** | **−** | **+** | ([Nicolas et al., 2005](#_ENREF_63); [Santiago et al., 2004](#_ENREF_81)) | Drug |
| Putatively: PFE0405c/PF3D7_0508200 or PF14_0034/PF3D7_1403700 | sphingosine N-acyltransferase | 2.3.1.24 | **−** | **−** | **+** | **−** | **+** | ([Gerold and Schwarz, 2001](#_ENREF_32)) | Drug |
| PFL2210w/PF3D7_1246100 | 5-aminolevulinate synthase | 2.3.1.37 | **−** | **−** | **+** | **+** | **+** | ([Surolia and Padmanaban, 1992](#_ENREF_90); [Varadharajan et al., 2002](#_ENREF_97)) | Drug |
| PFB0505c/PF3D7_0211400 \| PFF1275c/PF3D7_0626300 | beta-ketoacyl-ACP synthase | 2.3.1.41 | **+** | **+** | **+** | **−** | **+** | ([Lee et al., 2009](#_ENREF_50); [Vaughan et al., 2009](#_ENREF_98); [Waller et al., 1998](#_ENREF_101); [Waller et al., 2003](#_ENREF_102)) | Drug |
| PF14_0155/PF3D7_1415700 | serine C-palmitoyltransferase | 2.3.1.50 | **−** | **−** | **+** | **−** | **+** | ([Gerold and Schwarz, 2001](#_ENREF_32)) | Drug |
| PFE0660c/PF3D7_0513300 | purine nucleoside phosphorylase | 2.4.2.1 | **+** | **+** | **−** | **SGR** | **+** | ([Cassera et al., 2011](#_ENREF_18); [Kicska et al., 2002](#_ENREF_43)) | Drug |
| PFE0630c/PF3D7_0512700 | orotate phosphoribosyltransferase | 2.4.2.10 | **−** | **−** | **+** | **+** | **+** | ([Krungkrai et al., 2004](#_ENREF_47); [Seymour et al., 1994](#_ENREF_83)) | Drug |
| PFF1410c/PF3D7_0629100 | Nicotinate phosphoribosyltransferase | 2.4.2.11 | **−** | **+** | **−** | **−** | **−** | No evidence |  |
| PF10_0121/PF3D7_1012400 | hypoxanthine guanine phosphoribosyltransferase | 2.4.2.8 | **+** | **+** | **−** | **+** | **+** | ([Dawson et al., 1993](#_ENREF_26); [Li et al., 1999](#_ENREF_51)) | Drug |
| PF11_0295/PF3D7_1128400 | Geranyltranstransferase, dimethylallyltranstransferase | 2.5.1.10, 2.5.1.1 | **−** | **−** | **−** | **+** | **−** | ([Artz et al., 2011](#_ENREF_2)) | Drug |
| PF08_0095/PF3D7_0810800 | dihydropteroate synthase | 2.5.1.15 | **−** | **+** | **+** | **+** | **−** | ([Triglia et al., 1997](#_ENREF_93); [Zhang and Meshnick, 1991](#_ENREF_109)) | Drug |
| PF11_0301/PF3D7_1129000 | Spermidine synthase | 2.5.1.16 | **−** | **+** | **−** | **+** | **+** | ([Becker et al., 2010](#_ENREF_8); [Haider et al., 2005](#_ENREF_36)) | Drug |
| PF14_0187/PF3D7_1419300 | glutathione-S transferase | 2.5.1.18 | **−** | **+** | **+** | **−** | **−** | ([Fritz-Wolf et al., 2003](#_ENREF_30); [Harwaldt et al., 2002](#_ENREF_38); [Liebau et al., 2002](#_ENREF_52); [Perbandt et al., 2004](#_ENREF_68)) | Drug |
| PFB0280w/PF3D7_0206400 | 3-Phosphoshikimate 1-carboxyvinyltransferase | 2.5.1.19 | **−** | **+** | **+** | **+** | **−** | ([Roberts et al., 1998](#_ENREF_77)) | Drug |
| ORPHAN | Farnesyl-diphosphate famesyltransferase | 2.5.1.21 | **+** | **+** | **−** | **−** | **−** | No evidence |  |
| PF14_0125/PF3D7_1412600 | deoxyhypusine synthase | 2.5.1.46 | **−** | **+** | **+** | **−** | **−** | ([Specht et al., 2008](#_ENREF_88)) | Drug |
| ORPHAN | 3-deoxy-7-phosphoheptulonate synthase | 2.5.1.54 | **−** | **−** | **−** | **+** | **−** | No evidence |  |
| PFF0120w/PF3D7_0602500& PF11_0483/PF3D7_1147500 | protein farnesyltransferase | 2.5.1.58 (2.5.1.59) | **−** | **−** | **+** | **−** | **−** | ([Fletcher et al., 2008](#_ENREF_28)) | Drug |
| PFI1090w/PF3D7_0922200 | S-adenosylmethionine synthetase | 2.5.1.6 | **−** | **−** | **−** | **+** | **+** | No evidence |  |
| PFB0200c/PF3D7_0204500 | Aspartate transaminase, tyrosine transaminase, phenylalanine(histidine) transaminase | 2.6.1.1, 2.6.1.5, 2.6.1.58 | **−** | **−** | **−** | **+** | **−** | No evidence |  |
| PFI1100w/PF3D7_0922400 | aminodeoxychorismate synthase | 2.6.1.85 | **−** | **−** | **−** | **+** | **−** | No evidence |  |
| PFF1155w/PF3D7_0624000 | hexokinase | 2.7.1.1 | **−** | **+** | **−** | **−** | **+** | No evidence |  |
| PFE0150c/PF3D7_0503100 | 4-diphosphocytidyl-2C-methyl-D-erythritol kinase | 2.7.1.148 | **−** | **−** | **+** | **+** | **−** | No evidence |  |
| Putatively: PFI0650c/PF3D7_0913300 | NAD+ kinase | 2.7.1.23 | **−** | **−** | **−** | **+** | **−** | No evidence |  |
| PF14_0415/PF3D7_1443700 | Dephospho-CoA kinase | 2.7.1.24 | **−** | **−** | **−** | **+** | **−** | No evidence |  |
| PF14_0020/PF3D7_1401800 | choline kinase | 2.7.1.32 | **−** | **+** | **+** | **−** | **+** | ([Choubey et al., 2007](#_ENREF_22)) | Drug |
| PF10_0363/PF3D7_1037100 | Pyruvate kinase | 2.7.1.40 | **−** | **−** | **−** | **+** | **−** | No evidence |  |
| PFI1105w/PF3D7_0922500 | Phosphoglycerate kinase | 2.7.2.3 | **−** | **−** | **−** | **+** | **−** | No evidence |  |
| PFI1420w/PF3D7_0928900 | Guanylate kinase | 2.7.4.8 | **−** | **−** | **−** | **+** | **−** | No evidence |  |
| PFL2465c/PF3D7_1251300 | dTMP kinase | 2.7.4.9 | **−** | **+** | **−** | **+** | **−** | No evidence |  |
| PF13_0157/PF3D7_1327800 \| PF13_0143/PF3D7_1325100 | phosphoribosyl pyrophosphate synthase | 2.7.6.1 | **−** | **−** | **−** | **+** | **+** | No evidence |  |
| PFI1195c/PF3D7_0924300 | Thiamine diphosphokinase | 2.7.6.2 | **−** | **−** | **−** | **+** | **−** | No evidence |  |
| MAL13P1.86/PF3D7_1316600 | choline-phosphate cytidylyltransferase | 2.7.7.15 | **−** | **−** | **+** | **SGR** | **+** | No evidence |  |
| PF13_0159/PF3D7_1327600 | Nicotinate-nucleotide adenylyltransferase | 2.7.7.18 | **−** | **−** | **−** | **+** | **−** | ([Plata et al., 2010](#_ENREF_69)) |  |
| MAL13P1.292/PF3D7_1359100 | FAD synthetase, riboflavin kinase | 2.7.7.2, 2.7.1.26 | **−** | **+** | **+** | **+** | **−** | No evidence |  |
| PF07_0018/PF3D7_0704700 | Pantetheine-phosphate adenylyltransferase | 2.7.7.3 | **−** | **−** | **+** | **+** | **−** | No evidence |  |
| PF14_0097/PF3D7_1409900 | phosphatidate cytidylyltransferase | 2.7.7.41 | **−** | **−** | **−** | **SGR** | **−** | No evidence |  |
| multiple genes | RNA polymerase | 2.7.7.6 | **+** | **−** | **−** | **−** | **−** | ([Ke et al., 2012](#_ENREF_42)) |  |
| PFA0340w/PF3D7_0106900 | 2-C-methyl-D-erythritol 4-phosphate cytidylyltransferase | 2.7.7.60 | **−** | **−** | **+** | **+** | **−** | No evidence |  |
| multiple genes | DNA polymerase | 2.7.7.7 | **+** | **−** | **−** | **−** | **−** | No evidence |  |
| PFF1375c/PF3D7_0628300 | Ethanolaminephosphotransferase, diacylglycerol cholinephosphotransferase | 2.7.8.1, 2.7.8.2 | **−** | **−** | **−** | **SGR** | **−** | No evidence |  |
| MAL13P1.82/PF3D7_1315600 | CDP-diacylglycerol-inositol 3-phosphatidyltransferase | 2.7.8.11 | **−** | **+** | **−** | **SGR** | **−** | No evidence |  |
| PFF1215w/PF3D7_062510 | Sphingomyelin synthase | 2.7.8.27 | **−** | **−** | **−** | **SGR** | **−** | ([Lauer et al., 1995](#_ENREF_49)) |  |
| PFF1210w/PF3D7_0625000.1 | sphingomyelin synthase/phosphatidic acid phosphatase check the new annotation | 2.7.8.3 | **−** | **−** | **+** | **−** | **+** | ([Gerold and Schwarz, 2001](#_ENREF_32)) | Drug |
| PF14_0511/PF3D7_1453800 | 6-phosphogluconolactonase, glucose-6-phosphate dehydrogenase | 3.1.1.31, 1.1.1.49 | **−** | **−** | **−** | **SGR** | **−** | ([Crooke et al., 2006](#_ENREF_24); [Preuss et al., 2012](#_ENREF_71)) | RNAi - Drug |
| multiple genes | lysophospholipase | 3.1.1.5 | **+** | **−** | **−** | **−** | **−** | Zidovetzki R, Sherman IW, Prudhomme J, Parasitology. 1994 Apr;108 ( Pt 3):249-55 | Drug |
| MAL8P1.151/PF3D7_0802500 \| PF11_0122/PF3D7_1111600(endonuclease/exonuclease/phosphatase family protein) \| PF13_0285/PF3D7_1354200 | Inositol-1,4,5-trisphosphate 5-phosphase | 3.1.3.56 | **−** | **+** | **−** | **−** | **−** | ([Ogwan'g et al., 1993](#_ENREF_66)) | Drug |
| PFL1870c/PF3D7_1238600 | sphingomyelinase | 3.1.4.12 | **+** | **+** | **−** | **SGR** | **−** | ([Brand et al., 2008](#_ENREF_14); [Hanada et al., 2002](#_ENREF_37)) | Drug |
| multiple genes | 3',5'-cyclic-nucleotide phosphodiesterase | 3.1.4.17 | **−** | **+** | **−** | **−** | **−** | ([Taylor et al., 2008](#_ENREF_91)) | Genetic |
| PFE1050w/PF3D7_0520900 | S-adenosyl-L-homocysteine hydrolase | 3.3.1.1 | **+** | **+** | **+** | **−** | **+** | ([Bujnicki et al., 2003](#_ENREF_15); [Kitade et al., 1999](#_ENREF_44); [Shuto et al., 2002](#_ENREF_86)) | Drug |
| PFF1190c/PF3D7_0624700 \| PFI0535w/PF3D7_0911000 | N-acetylglucosaminylphosphotidylinositol deacetylase | 3.5.1.89 | **−** | **−** | **+** | **−** | **−** | No evidence |  |
| PF14_0697/PF3D7_1472900 | dihydroorotase | 3.5.2.3 | **−** | **−** | **+** | **+** | **+** | ([Krungkrai et al., 1992](#_ENREF_46); [Seymour et al., 1997](#_ENREF_84)) | Drug |
| PF13_0259/PF3D7_1349400 | dCTP deaminase | 3.5.4.13 | **−** | **−** | **−** | **SGR** | **−** | No evidence |  |
| PF10_0289/PF3D7_1029600 | adenosine deaminase | 3.5.4.4 | **−** | **+** | **−** | **+** | **+** | ([Ho et al., 2009](#_ENREF_39); [Tyler et al., 2007](#_ENREF_94)) | Drug |
| PFE1035c/PF3D7_0520600 | bis(5'-nucleosyl)-tetraphosphatase | 3.6.1.17 (3.6.1.41) | **−** | **+** | **−** | **−** | **−** | No evidence |  |
| PFF0370w/PF3D7_0607500 | 3-octaprenyl-4-hydroxybenzoate carboxy-lyase | 4.1.1.- | **−** | **−** | **−** | **+** | **−** | No evidence |  |
| ORPHAN | 3-octaprenyl-4-hydroxybenzoate carboxy-lyase | 4.1.1.- | **−** | **−** | **−** | **+** | **−** | No evidence |  |
| PF10_0322/PF3D7_1033100 | ornithine decarboxylase | 4.1.1.17 | **−** | **+** | **−** | **+** | **+** | ([Assaraf et al., 1984](#_ENREF_3); [Das Gupta et al., 2005](#_ENREF_25); [Muller et al., 2008](#_ENREF_59)) | Drug |
| PF10_0225/PF3D7_1023200 | orotidine-5'-phosphate decarboxylase | 4.1.1.23 | **−** | **+** | **+** | **+** | **+** | ([Krungkrai et al., 2005](#_ENREF_48); [Meza-Avina et al., 2008](#_ENREF_58); [Scott et al., 1986](#_ENREF_82)) | Drug |
| MAL8P1.81/PF3D7_0816100 | Phosphopantothenoylcysteine decarboxylase | 4.1.1.36 | **−** | **−** | **−** | **+** | **−** | No evidence |  |
| PFF0360w/PF3D7_0607300 | Uroporphyrinogen decarboxylase | 4.1.1.37 | **−** | **−** | **+** | **+** | **−** | No evidence |  |
| PF10_0322/PF3D7_1033100 | S-adenosylmethionine decarboxylase | 4.1.1.50 | **+** | **+** | **−** | **+** | **+** | ([Das Gupta et al., 2005](#_ENREF_25); [Muller et al., 2008](#_ENREF_59); [Ramya et al., 2006](#_ENREF_74); [Wright et al., 1991](#_ENREF_107)) | Drug |
| PF14_0425/PF3D7_1444800 | aldolase | 4.1.2.13 | **+** | **+** | **−** | **GR** | **+** | ([Wanidworanun et al., 1999](#_ENREF_104)) | antisense ODN |
| ORPHAN | Chorismate lyase | 4.1.3.40 | **−** | **−** | **−** | **+** | **−** | No evidence |  |
| PF11_0411/PF3D7_1140000 (PF11_0410/PF3D7_1140000) | carbonate dehydratase | 4.2.1.1 | **−** | **−** | **+** | **+** | **+** | ([Reungprapavut et al., 2004](#_ENREF_76)) | Drug |
| ORPHAN | 3-dehydroquinate dehydratase | 4.2.1.10 | **−** | **−** | **−** | **+** | **−** | No evidence |  |
| PF10_0155/PF3D7_1015900 | Phosphopyruvate hydratase / enolase | 4.2.1.11 | **−** | **−** | **−** | **+** | **−** | ([Ghosh et al., 2011](#_ENREF_33))8. | Antibody |
| PF14_0381/PF3D7_1440300 | delta-aminolevulinate dehydratase | 4.2.1.24 | **−** | **+** | **+** | **+** | **+** | ([Bonday et al., 2000](#_ENREF_13); [Ramya et al., 2007](#_ENREF_73)) | Drug |
| PF13_0128/PF3D7_1323000 | 3-hydroxyacyl-ACP dehydratase | 4.2.1.58 – 61 | **−** | **−** | **+** | **+** | **−** | ([Sharma et al., 2003](#_ENREF_85); [Vaughan et al., 2009](#_ENREF_98)) | Drug - Genetic |
| PFL0480w/PF3D7_1209600 | Uroporphyrinogen-III synthase | 4.2.1.75 | **−** | **−** | **−** | **+** | **−** | No evidence (reported by Plata et al as an orpahn we assign gene based on PMID: 17962188) |  |
| ORPHAN | 3-dehydroquinate synthase | 4.2.3.4 | **−** | **−** | **−** | **+** | **−** | No evidence |  |
| PFF1105c/PF3D7_0623000 | chorismate synthase | 4.2.3.5 | **+** | **+** | **−** | **+** | **−** | ([McRobert and McConkey, 2002](#_ENREF_56)) | RNAi |
| PFB0295w/PF3D7_0206700 | adenylosuccinate lyase | 4.3.2.2 | **−** | **−** | **−** | **+** | **+** | ([Bulusu et al., 2009](#_ENREF_16)) | Drug |
| PFF0230c/PF3D7_0604700 \| PF11_0145/PF3D7_1113700 | glyoxylase | 4.4.1.5 | **+** | **+** | **+** | **−** | **−** | ([Thornalley et al., 1994](#_ENREF_92); [Urscher et al., 2012](#_ENREF_95)) | Drug |
| PFB0420w/PF3D7_0209300 | 2C-methyl-D-erythritol 2,4-cyclodiphosphate synthase | 4.6.1.12 | **−** | **−** | **+** | **+** | **−** | ([Crane et al., 2006](#_ENREF_23)) | Drug |
| MAL13P1.326/PF3D7_1364900 | Ferrochelatase | 4.99.1.1 | **−** | **−** | **−** | **+** | **−** | No evidence |  |
| PFL0960w/PF3D7_1219900 | Ribulose-phosphate 3-epimerase | 5.1.3.1 | **−** | **−** | **−** | **GR** | **−** | No evidence |  |
| PF14_0378 / PFC0831w | triosephosphate isomerase | 5.3.1.1 | **−** | **−** | **−** | **GR** | **+** | ([Barker et al., 1996](#_ENREF_7)) | antisense oligodeoxynucleotides |
| PF14_0341/PF3D7_1436000 | Glucose-6-phosphate isomerase | 5.3.1.9 | **−** | **−** | **−** | **SGR** | **−** | No evidence |  |
| PF10_0122/PF3D7_1012500 | Phosphoglucomutase | 5.4.2.2 | **−** | **−** | **−** | **SGR** | **−** | No evidence |  |
| PFE0520c/PF3D7_0510500 | topoisomerase I | 5.99.1.2 | **−** | **−** | **+** | **−** | **−** | ([Bodley et al., 1998](#_ENREF_12)) | Drug |
| PFE0520c/PF3D7_0510500 \| PF13_0251/PF3D7_1347100 | topoisomerase II | 5.99.1.3 | **−** | **−** | **+** | **−** | **−** | ([Chavalitshewinkoon-Petmitr et al., 2000](#_ENREF_21); [Gamage et al., 1994](#_ENREF_31); [Noonpakdee et al., 2003](#_ENREF_64)) | Drug |
| PF11_0270/PF3D7_1126000 | threonine-tRNA ligase | 6.1.1.3 | **−** | **+** | **+** | **−** | **−** | No evidence |  |
| PF13_0354/PF3D7_1367700 | alanine-tRNA ligase | 6.1.1.7 | **−** | **+** | **+** | **−** | **−** | No evidence |  |
| PFF1350c/PF3D7_0627800 | acetyl-CoA synthetase | 6.2.1.1 | **−** | **−** | **−** | **−** | **+** | No evidence |  |
| multiple genes | acyl-CoA synthetase | 6.2.1.3 | **−** | **+** | **−** | **−** | **+** | No evidence |  |
| PF13_0140/PF3D7_1324800 | dihydrofolate synthase | 6.3.2.12 | **−** | **−** | **−** | **+** | **−** | ([Salcedo et al., 2001](#_ENREF_78)) | Drug |
| PF13_0140/PF3D7_1324800 | folylpolyglutamate synthase | 6.3.2.17 | **−** | **−** | **−** | **+** | **+** | ([Salcedo et al., 2001](#_ENREF_78); [Wang et al., 2010](#_ENREF_103)) | Drug |
| PFI0925w/PF3D7_0918900 | gamma-glutamylcysteine synthetase | 6.3.2.2 | **+** | **+** | **+** | **−** | **+** | ([Luersen et al., 2000](#_ENREF_53); [Meierjohann et al., 2002](#_ENREF_57); [Platel et al., 1999](#_ENREF_70)) | Drug |
| PF14_0100/PF3D7_1410200 | CTP synthase | 6.3.4.2 | **−** | **−** | **−** | **−** | **+** | No evidence |  |
| PF13_0287/PF3D7_1354500 | adenylosuccinate synthase | 6.3.4.4 | **−** | **−** | **+** | **+** | **+** | ([Raman et al., 2004](#_ENREF_72)) |  |
| PFI1310w/PF3D7_0926700 | NAD+ synthase (glutamine-hydrolysing) | 6.3.5.1 | **−** | **−** | **−** | **+** | **−** | No evidence |  |
| PF10_0123/PF3D7_1012600 | GMP synthase | 6.3.5.2 | **−** | **−** | **−** | **−** | **+** | ([McConkey, 2000](#_ENREF_55)) |  |
| PF13_0044/PF3D7_1308200 | carbamoyl phosphate synthetase | 6.3.5.5 | **+** | **+** | **+** | **+** | **+** | ([Flores et al., 1997](#_ENREF_29)) | Ribo -zyme |
| PF10_0409/PF3D7_1026900 \| PF14_0664/PF3D7_1469600 | acetyl-CoA carboxylase | 6.4.1.2 | **−** | **−** | **+** | **−** | **+** | ([Waller et al., 2003](#_ENREF_102)) | Drug |
| multiple genes | histone deacetylase | none | **+** | **−** | **−** | **−** | **−** | ([Andrews et al., 2012](#_ENREF_1); [Sumanadasa et al., 2012](#_ENREF_89)) | Drug |

**Supplementary References**

Andrews, K.T., Tran, T.N., and Fairlie, D.P. (2012). Towards histone deacetylase inhibitors as new antimalarial drugs. Current pharmaceutical design *18*, 3467-3479.

Artz, J.D., Wernimont, A.K., Dunford, J.E., Schapira, M., Dong, A., Zhao, Y., Lew, J., Russell, R.G., Ebetino, F.H., Oppermann, U., et al. (2011). Molecular characterization of a novel geranylgeranyl pyrophosphate synthase from Plasmodium parasites. The Journal of biological chemistry *286*, 3315-3322.

Assaraf, Y.G., Golenser, J., Spira, D.T., and Bachrach, U. (1984). Polyamine levels and the activity of their biosynthetic enzymes in human erythrocytes infected with the malarial parasite, Plasmodium falciparum. The Biochemical journal *222*, 815-819.

Baldwin, J., Michnoff, C.H., Malmquist, N.A., White, J., Roth, M.G., Rathod, P.K., and Phillips, M.A. (2005). High-throughput screening for potent and selective inhibitors of Plasmodium falciparum dihydroorotate dehydrogenase. The Journal of biological chemistry *280*, 21847-21853.

Banerjee, A.K., Arora, N., and Murty, U.S.N. (2012a). Aspartate carbamoyltransferase of Plasmodium falciparum as a potential drug target for designing anti-malarial chemotherapeutic agents. Medicinal Chemistry Research *21*, 2480-2493.

Banerjee, T., Jaijyan, D.K., Surolia, N., Singh, A.P., and Surolia, A. (2012b). Apicoplast triose phosphate transporter (TPT) gene knockout is lethal for Plasmodium. Molecular and biochemical parasitology *186*, 44-50.

Barker, R.H., Jr., Metelev, V., Rapaport, E., and Zamecnik, P. (1996). Inhibition of Plasmodium falciparum malaria using antisense oligodeoxynucleotides. Proceedings of the National Academy of Sciences of the United States of America *93*, 514-518.

Becker, J.V., Mtwisha, L., Crampton, B.G., Stoychev, S., van Brummelen, A.C., Reeksting, S., Louw, A.I., Birkholtz, L.M., and Mancama, D.T. (2010). Plasmodium falciparum spermidine synthase inhibition results in unique perturbation-specific effects observed on transcript, protein and metabolite levels. BMC genomics *11*, 235.

Biagini, G.A., Fisher, N., Shone, A.E., Mubaraki, M.A., Srivastava, A., Hill, A., Antoine, T., Warman, A.J., Davies, J., Pidathala, C., et al. (2012). Generation of quinolone antimalarials targeting the Plasmodium falciparum mitochondrial respiratory chain for the treatment and prophylaxis of malaria. Proceedings of the National Academy of Sciences of the United States of America *109*, 8298-8303.

Biagini, G.A., Viriyavejakul, P., O'Neill P, M., Bray, P.G., and Ward, S.A. (2006). Functional characterization and target validation of alternative complex I of Plasmodium falciparum mitochondria. Antimicrobial agents and chemotherapy *50*, 1841-1851.

Biot, C., Bauer, H., Schirmer, R.H., and Davioud-Charvet, E. (2004). 5-substituted tetrazoles as bioisosteres of carboxylic acids. Bioisosterism and mechanistic studies on glutathione reductase inhibitors as antimalarials. Journal of medicinal chemistry *47*, 5972-5983.

Bodley, A.L., Cumming, J.N., and Shapiro, T.A. (1998). Effects of camptothecin, a topoisomerase I inhibitor, on Plasmodium falciparum. Biochemical pharmacology *55*, 709-711.

Bonday, Z.Q., Dhanasekaran, S., Rangarajan, P.N., and Padmanaban, G. (2000). Import of host delta-aminolevulinate dehydratase into the malarial parasite: identification of a new drug target. Nature medicine *6*, 898-903.

Brand, V., Koka, S., Lang, C., Jendrossek, V., Huber, S.M., Gulbins, E., and Lang, F. (2008). Influence of amitriptyline on eryptosis, parasitemia and survival of Plasmodium berghei-infected mice. Cellular physiology and biochemistry : international journal of experimental cellular physiology, biochemistry, and pharmacology *22*, 405-412.

Bujnicki, J.M., Prigge, S.T., Caridha, D., and Chiang, P.K. (2003). Structure, evolution, and inhibitor interaction of S-adenosyl-L-homocysteine hydrolase from Plasmodium falciparum. Proteins *52*, 624-632.

Bulusu, V., Srinivasan, B., Bopanna, M.P., and Balaram, H. (2009). Elucidation of the substrate specificity, kinetic and catalytic mechanism of adenylosuccinate lyase from Plasmodium falciparum. Biochimica et biophysica acta *1794*, 642-654.

Cassera, M.B., Gozzo, F.C., D'Alexandri, F.L., Merino, E.F., del Portillo, H.A., Peres, V.J., Almeida, I.C., Eberlin, M.N., Wunderlich, G., Wiesner, J., et al. (2004). The methylerythritol phosphate pathway is functionally active in all intraerythrocytic stages of Plasmodium falciparum. The Journal of biological chemistry *279*, 51749-51759.

Cassera, M.B., Hazleton, K.Z., Merino, E.F., Obaldia, N., 3rd, Ho, M.C., Murkin, A.S., DePinto, R., Gutierrez, J.A., Almo, S.C., Evans, G.B., et al. (2011). Plasmodium falciparum parasites are killed by a transition state analogue of purine nucleoside phosphorylase in a primate animal model. PloS one *6*, e26916.

Cassera, M.B., Merino, E.F., Peres, V.J., Kimura, E.A., Wunderlich, G., and Katzin, A.M. (2007). Effect of fosmidomycin on metabolic and transcript profiles of the methylerythritol phosphate pathway in Plasmodium falciparum. Memorias do Instituto Oswaldo Cruz *102*, 377-383.

Chakrabarti, D., Schuster, S.M., and Chakrabarti, R. (1993). Cloning and characterization of subunit genes of ribonucleotide reductase, a cell-cycle-regulated enzyme, from Plasmodium falciparum. Proceedings of the National Academy of Sciences of the United States of America *90*, 12020-12024.

Chavalitshewinkoon-Petmitr, P., Pongvilairat, G., Auparakkitanon, S., and Wilairat, P. (2000). Gametocytocidal activity of pyronaridine and DNA topoisomerase II inhibitors against multidrug-resistant Plasmodium falciparum in vitro. Parasitology international *48*, 275-280.

Choubey, V., Maity, P., Guha, M., Kumar, S., Srivastava, K., Puri, S.K., and Bandyopadhyay, U. (2007). Inhibition of Plasmodium falciparum choline kinase by hexadecyltrimethylammonium bromide: a possible antimalarial mechanism. Antimicrobial agents and chemotherapy *51*, 696-706.

Crane, C.M., Kaiser, J., Ramsden, N.L., Lauw, S., Rohdich, F., Eisenreich, W., Hunter, W.N., Bacher, A., and Diederich, F. (2006). Fluorescent inhibitors for IspF, an enzyme in the non-mevalonate pathway for isoprenoid biosynthesis and a potential target for antimalarial therapy. Angewandte Chemie *45*, 1069-1074.

Crooke, A., Diez, A., Mason, P.J., and Bautista, J.M. (2006). Transient silencing of Plasmodium falciparum bifunctional glucose-6-phosphate dehydrogenase- 6-phosphogluconolactonase. The FEBS journal *273*, 1537-1546.

Das Gupta, R., Krause-Ihle, T., Bergmann, B., Muller, I.B., Khomutov, A.R., Muller, S., Walter, R.D., and Luersen, K. (2005). 3-Aminooxy-1-aminopropane and derivatives have an antiproliferative effect on cultured Plasmodium falciparum by decreasing intracellular polyamine concentrations. Antimicrobial agents and chemotherapy *49*, 2857-2864.

Dawson, P.A., Cochran, D.A., Emmerson, B.T., and Gordon, R.B. (1993). Inhibition of Plasmodium falciparum hypoxanthine-guanine phosphoribosyltransferase mRNA by antisense oligodeoxynucleotide sequence. Molecular and biochemical parasitology *60*, 153-156.

Deng, X., Gujjar, R., El Mazouni, F., Kaminsky, W., Malmquist, N.A., Goldsmith, E.J., Rathod, P.K., and Phillips, M.A. (2009). Structural plasticity of malaria dihydroorotate dehydrogenase allows selective binding of diverse chemical scaffolds. The Journal of biological chemistry *284*, 26999-27009.

Fletcher, S., Cummings, C.G., Rivas, K., Katt, W.P., Horney, C., Buckner, F.S., Chakrabarti, D., Sebti, S.M., Gelb, M.H., Van Voorhis, W.C., et al. (2008). Potent, Plasmodium-selective farnesyltransferase inhibitors that arrest the growth of malaria parasites: structure-activity relationships of ethylenediamine-analogue scaffolds and homology model validation. Journal of medicinal chemistry *51*, 5176-5197.

Flores, M.V., Atkins, D., Wade, D., O'Sullivan, W.J., and Stewart, T.S. (1997). Inhibition of Plasmodium falciparum proliferation in vitro by ribozymes. The Journal of biological chemistry *272*, 16940-16945.

Fritz-Wolf, K., Becker, A., Rahlfs, S., Harwaldt, P., Schirmer, R.H., Kabsch, W., and Becker, K. (2003). X-ray structure of glutathione S-transferase from the malarial parasite Plasmodium falciparum. Proceedings of the National Academy of Sciences of the United States of America *100*, 13821-13826.

Gamage, S.A., Tepsiri, N., Wilairat, P., Wojcik, S.J., Figgitt, D.P., Ralph, R.K., and Denny, W.A. (1994). Synthesis and in vitro evaluation of 9-anilino-3,6-diaminoacridines active against a multidrug-resistant strain of the malaria parasite Plasmodium falciparum. Journal of medicinal chemistry *37*, 1486-1494.

Gerold, P., and Schwarz, R.T. (2001). Biosynthesis of glycosphingolipids de-novo by the human malaria parasite Plasmodium falciparum. Molecular and biochemical parasitology *112*, 29-37.

Ghosh, A.K., Coppens, I., Gardsvoll, H., Ploug, M., and Jacobs-Lorena, M. (2011). Plasmodium ookinetes coopt mammalian plasminogen to invade the mosquito midgut. Proceedings of the National Academy of Sciences of the United States of America *108*, 17153-17158.

Grellier, P., Maroziene, A., Nivinskas, H., Sarlauskas, J., Aliverti, A., and Cenas, N. (2010). Antiplasmodial activity of quinones: roles of aziridinyl substituents and the inhibition of Plasmodium falciparum glutathione reductase. Archives of biochemistry and biophysics *494*, 32-39.

Gujjar, R., El Mazouni, F., White, K.L., White, J., Creason, S., Shackleford, D.M., Deng, X., Charman, W.N., Bathurst, I., Burrows, J., et al. (2011). Lead optimization of aryl and aralkyl amine-based triazolopyrimidine inhibitors of Plasmodium falciparum dihydroorotate dehydrogenase with antimalarial activity in mice. Journal of medicinal chemistry *54*, 3935-3949.

Haider, N., Eschbach, M.L., Dias Sde, S., Gilberger, T.W., Walter, R.D., and Luersen, K. (2005). The spermidine synthase of the malaria parasite Plasmodium falciparum: molecular and biochemical characterisation of the polyamine synthesis enzyme. Molecular and biochemical parasitology *142*, 224-236.

Hanada, K., Palacpac, N.M., Magistrado, P.A., Kurokawa, K., Rai, G., Sakata, D., Hara, T., Horii, T., Nishijima, M., and Mitamura, T. (2002). Plasmodium falciparum phospholipase C hydrolyzing sphingomyelin and lysocholinephospholipids is a possible target for malaria chemotherapy. The Journal of experimental medicine *195*, 23-34.

Harwaldt, P., Rahlfs, S., and Becker, K. (2002). Glutathione S-transferase of the malarial parasite Plasmodium falciparum: characterization of a potential drug target. Biological chemistry *383*, 821-830.

Ho, M.C., Cassera, M.B., Madrid, D.C., Ting, L.M., Tyler, P.C., Kim, K., Almo, S.C., and Schramm, V.L. (2009). Structural and metabolic specificity of methylthiocoformycin for malarial adenosine deaminases. Biochemistry *48*, 9618-9626.

Jiang, L., Lee, P.C., White, J., and Rathod, P.K. (2000). Potent and selective activity of a combination of thymidine and 1843U89, a folate-based thymidylate synthase inhibitor, against Plasmodium falciparum. Antimicrobial agents and chemotherapy *44*, 1047-1050.

Jomaa, H., Wiesner, J., Sanderbrand, S., Altincicek, B., Weidemeyer, C., Hintz, M., Turbachova, I., Eberl, M., Zeidler, J., Lichtenthaler, H.K., et al. (1999). Inhibitors of the nonmevalonate pathway of isoprenoid biosynthesis as antimalarial drugs. Science *285*, 1573-1576.

Ke, H., Morrisey, J.M., Ganesan, S.M., Mather, M.W., and Vaidya, A.B. (2012). Mitochondrial RNA polymerase is an essential enzyme in erythrocytic stages of Plasmodium falciparum. Molecular and biochemical parasitology *185*, 48-51.

Kicska, G.A., Tyler, P.C., Evans, G.B., Furneaux, R.H., Schramm, V.L., and Kim, K. (2002). Purine-less death in Plasmodium falciparum induced by immucillin-H, a transition state analogue of purine nucleoside phosphorylase. The Journal of biological chemistry *277*, 3226-3231.

Kitade, Y., Kozaki, A., Gotoh, T., Miwa, T., Nakanishi, M., and Yatome, C. (1999). Synthesis of S-adenosyl-L-homocysteine hydrolase inhibitors and their biological activities. Nucleic acids symposium series, 25-26.

Krnajski, Z., Gilberger, T.W., Walter, R.D., Cowman, A.F., and Muller, S. (2002). Thioredoxin reductase is essential for the survival of Plasmodium falciparum erythrocytic stages. The Journal of biological chemistry *277*, 25970-25975.

Krungkrai, J., Krungkrai, S.R., and Phakanont, K. (1992). Antimalarial activity of orotate analogs that inhibit dihydroorotase and dihydroorotate dehydrogenase. Biochemical pharmacology *43*, 1295-1301.

Krungkrai, S.R., Aoki, S., Palacpac, N.M., Sato, D., Mitamura, T., Krungkrai, J., and Horii, T. (2004). Human malaria parasite orotate phosphoribosyltransferase: functional expression, characterization of kinetic reaction mechanism and inhibition profile. Molecular and biochemical parasitology *134*, 245-255.

Krungkrai, S.R., DelFraino, B.J., Smiley, J.A., Prapunwattana, P., Mitamura, T., Horii, T., and Krungkrai, J. (2005). A novel enzyme complex of orotate phosphoribosyltransferase and orotidine 5'-monophosphate decarboxylase in human malaria parasite Plasmodium falciparum: physical association, kinetics, and inhibition characterization. Biochemistry *44*, 1643-1652.

Lauer, S.A., Ghori, N., and Haldar, K. (1995). Sphingolipid synthesis as a target for chemotherapy against malaria parasites. Proceedings of the National Academy of Sciences of the United States of America *92*, 9181-9185.

Lee, P.J., Bhonsle, J.B., Gaona, H.W., Huddler, D.P., Heady, T.N., Kreishman-Deitrick, M., Bhattacharjee, A., McCalmont, W.F., Gerena, L., Lopez-Sanchez, M., et al. (2009). Targeting the fatty acid biosynthesis enzyme, beta-ketoacyl-acyl carrier protein synthase III (PfKASIII), in the identification of novel antimalarial agents. Journal of medicinal chemistry *52*, 952-963.

Li, C.M., Tyler, P.C., Furneaux, R.H., Kicska, G., Xu, Y., Grubmeyer, C., Girvin, M.E., and Schramm, V.L. (1999). Transition-state analogs as inhibitors of human and malarial hypoxanthine-guanine phosphoribosyltransferases. Nature structural biology *6*, 582-587.

Liebau, E., Bergmann, B., Campbell, A.M., Teesdale-Spittle, P., Brophy, P.M., Luersen, K., and Walter, R.D. (2002). The glutathione S-transferase from Plasmodium falciparum. Molecular and biochemical parasitology *124*, 85-90.

Luersen, K., Walter, R.D., and Muller, S. (2000). Plasmodium falciparum-infected red blood cells depend on a functional glutathione de novo synthesis attributable to an enhanced loss of glutathione. The Biochemical journal *346 Pt 2*, 545-552.

Massimine, K.M., McIntosh, M.T., Doan, L.T., Atreya, C.E., Gromer, S., Sirawaraporn, W., Elliott, D.A., Joiner, K.A., Schirmer, R.H., and Anderson, K.S. (2006). Eosin B as a novel antimalarial agent for drug-resistant Plasmodium falciparum. Antimicrobial agents and chemotherapy *50*, 3132-3141.

McConkey, G.A. (2000). Plasmodium falciparum: isolation and characterisation of a gene encoding protozoan GMP synthase. Experimental parasitology *94*, 23-32.

McRobert, L., and McConkey, G.A. (2002). RNA interference (RNAi) inhibits growth of Plasmodium falciparum. Molecular and biochemical parasitology *119*, 273-278.

Meierjohann, S., Walter, R.D., and Muller, S. (2002). Regulation of intracellular glutathione levels in erythrocytes infected with chloroquine-sensitive and chloroquine-resistant Plasmodium falciparum. The Biochemical journal *368*, 761-768.

Meza-Avina, M.E., Wei, L., Buhendwa, M.G., Poduch, E., Bello, A.M., Pai, E.F., and Kotra, L.P. (2008). Inhibition of orotidine 5'-monophosphate decarboxylase and its therapeutic potential. Mini reviews in medicinal chemistry *8*, 239-247.

Muller, I.B., Das Gupta, R., Luersen, K., Wrenger, C., and Walter, R.D. (2008). Assessing the polyamine metabolism of Plasmodium falciparum as chemotherapeutic target. Molecular and biochemical parasitology *160*, 1-7.

Munro, J.B., and Silva, J.C. (2012). Ribonucleotide reductase as a target to control apicomplexan diseases. Current issues in molecular biology *14*, 9-26.

Nagaraj, V.A., Arumugam, R., Prasad, D., Rangarajan, P.N., and Padmanaban, G. (2010). Protoporphyrinogen IX oxidase from Plasmodium falciparum is anaerobic and is localized to the mitochondrion. Molecular and biochemical parasitology *174*, 44-52.

Nduati, E., Hunt, S., Kamau, E.M., and Nzila, A. (2005). 2,4-diaminopteridine-based compounds as precursors for de novo synthesis of antifolates: a novel class of antimalarials. Antimicrobial agents and chemotherapy *49*, 3652-3657.

Nicolas, O., Margout, D., Taudon, N., Wein, S., Calas, M., Vial, H.J., and Bressolle, F.M. (2005). Pharmacological properties of a new antimalarial bisthiazolium salt, T3, and a corresponding prodrug, TE3. Antimicrobial agents and chemotherapy *49*, 3631-3639.

Noonpakdee, W., Pothikasikorn, J., Nimitsantiwong, W., and Wilairat, P. (2003). Inhibition of Plasmodium falciparum proliferation in vitro by antisense oligodeoxynucleotides against malarial topoisomerase II. Biochemical and biophysical research communications *302*, 659-664.

Odom, A.R., and Van Voorhis, W.C. (2010). Functional genetic analysis of the Plasmodium falciparum deoxyxylulose 5-phosphate reductoisomerase gene. Molecular and biochemical parasitology *170*, 108-111.

Ogwan'g, R., Mwangi, J., Gachihi, G., Nwachukwu, A., Roberts, C.R., and Martin, S.K. (1993). Use of pharmacological agents to implicate a role for phosphoinositide hydrolysis products in malaria gamete formation. Biochemical pharmacology *46*, 1601-1606.

Painter, H.J., Morrisey, J.M., Mather, M.W., and Vaidya, A.B. (2007). Specific role of mitochondrial electron transport in blood-stage Plasmodium falciparum. Nature *446*, 88-91.

Perbandt, M., Burmeister, C., Walter, R.D., Betzel, C., and Liebau, E. (2004). Native and inhibited structure of a Mu class-related glutathione S-transferase from Plasmodium falciparum. The Journal of biological chemistry *279*, 1336-1342.

Plata, G., Hsiao, T.L., Olszewski, K.L., Llinas, M., and Vitkup, D. (2010). Reconstruction and flux-balance analysis of the Plasmodium falciparum metabolic network. Molecular systems biology *6*, 408.

Platel, D.F., Mangou, F., and Tribouley-Duret, J. (1999). Role of glutathione in the detoxification of ferriprotoporphyrin IX in chloroquine resistant Plasmodium berghei. Molecular and biochemical parasitology *98*, 215-223.

Preuss, J., Maloney, P., Peddibhotla, S., Hedrick, M.P., Hershberger, P., Gosalia, P., Milewski, M., Li, Y.L., Sugarman, E., Hood, B., et al. (2012). Discovery of a Plasmodium falciparum glucose-6-phosphate dehydrogenase 6-phosphogluconolactonase inhibitor (R,Z)-N-((1-ethylpyrrolidin-2-yl)methyl)-2-(2-fluorobenzylidene)-3-oxo-3,4-dihydr o-2H-benzo[b][1,4]thiazine-6-carboxamide (ML276) that reduces parasite growth in vitro. Journal of medicinal chemistry *55*, 7262-7272.

Raman, J., Mehrotra, S., Anand, R.P., and Balaram, H. (2004). Unique kinetic mechanism of Plasmodium falciparum adenylosuccinate synthetase. Molecular and biochemical parasitology *138*, 1-8.

Ramya, T.N., Mishra, S., Karmodiya, K., Surolia, N., and Surolia, A. (2007). Inhibitors of nonhousekeeping functions of the apicoplast defy delayed death in Plasmodium falciparum. Antimicrobial agents and chemotherapy *51*, 307-316.

Ramya, T.N., Surolia, N., and Surolia, A. (2006). Polyamine synthesis and salvage pathways in the malaria parasite Plasmodium falciparum. Biochemical and biophysical research communications *348*, 579-584.

Razakantoanina, V., Nguyen Kim, P.P., and Jaureguiberry, G. (2000). Antimalarial activity of new gossypol derivatives. Parasitology research *86*, 665-668.

Reungprapavut, S., Krungkrai, S.R., and Krungkrai, J. (2004). Plasmodium falciparum carbonic anhydrase is a possible target for malaria chemotherapy. Journal of enzyme inhibition and medicinal chemistry *19*, 249-256.

Roberts, F., Roberts, C.W., Johnson, J.J., Kyle, D.E., Krell, T., Coggins, J.R., Coombs, G.H., Milhous, W.K., Tzipori, S., Ferguson, D.J., et al. (1998). Evidence for the shikimate pathway in apicomplexan parasites. Nature *393*, 801-805.

Salcedo, E., Cortese, J.F., Plowe, C.V., Sims, P.F., and Hyde, J.E. (2001). A bifunctional dihydrofolate synthetase--folylpolyglutamate synthetase in Plasmodium falciparum identified by functional complementation in yeast and bacteria. Molecular and biochemical parasitology *112*, 239-252.

Saliba, K.J., Horner, H.A., and Kirk, K. (1998). Transport and metabolism of the essential vitamin pantothenic acid in human erythrocytes infected with the malaria parasite Plasmodium falciparum. The Journal of biological chemistry *273*, 10190-10195.

Saliba, K.J., Martin, R.E., Broer, A., Henry, R.I., McCarthy, C.S., Downie, M.J., Allen, R.J., Mullin, K.A., McFadden, G.I., Broer, S., et al. (2006). Sodium-dependent uptake of inorganic phosphate by the intracellular malaria parasite. Nature *443*, 582-585.

Santiago, T.C., Zufferey, R., Mehra, R.S., Coleman, R.A., and Mamoun, C.B. (2004). The Plasmodium falciparum PfGatp is an endoplasmic reticulum membrane protein important for the initial step of malarial glycerolipid synthesis. The Journal of biological chemistry *279*, 9222-9232.

Scott, H.V., Gero, A.M., and O'Sullivan, W.J. (1986). In vitro inhibition of Plasmodium falciparum by pyrazofurin, an inhibitor of pyrimidine biosynthesis de novo. Molecular and biochemical parasitology *18*, 3-15.

Seymour, K.K., Lyons, S.D., Phillips, L., Rieckmann, K.H., and Christopherson, R.I. (1994). Cytotoxic effects of inhibitors of de novo pyrimidine biosynthesis upon Plasmodium falciparum. Biochemistry *33*, 5268-5274.

Seymour, K.K., Yeo, A.E., Rieckmann, K.H., and Christopherson, R.I. (1997). dCTP levels are maintained in Plasmodium falciparum subjected to pyrimidine deficiency or excess. Annals of tropical medicine and parasitology *91*, 603-609.

Sharma, S.K., Kapoor, M., Ramya, T.N., Kumar, S., Kumar, G., Modak, R., Sharma, S., Surolia, N., and Surolia, A. (2003). Identification, characterization, and inhibition of Plasmodium falciparum beta-hydroxyacyl-acyl carrier protein dehydratase (FabZ). The Journal of biological chemistry *278*, 45661-45671.

Shuto, S., Minakawa, N., Niizuma, S., Kim, H.S., Wataya, Y., and Matsuda, A. (2002). New neplanocin analogues. 12. Alternative synthesis and antimalarial effect of (6'R)-6'-C-methylneplanocin A, a potent AdoHcy hydrolase inhibitor. Journal of medicinal chemistry *45*, 748-751.

Soulere, L., Delplace, P., Davioud-Charvet, E., Py, S., Sergheraert, C., Perie, J., Ricard, I., Hoffmann, P., and Dive, D. (2003). Screening of Plasmodium falciparum iron superoxide dismutase inhibitors and accuracy of the SOD-assays. Bioorganic & medicinal chemistry *11*, 4941-4944.

Specht, S., Sarite, S.R., Hauber, I., Hauber, J., Gorbig, U.F., Meier, C., Bevec, D., Hoerauf, A., and Kaiser, A. (2008). The guanylhydrazone CNI-1493: an inhibitor with dual activity against malaria-inhibition of host cell pro-inflammatory cytokine release and parasitic deoxyhypusine synthase. Parasitology research *102*, 1177-1184.

Sumanadasa, S.D., Goodman, C.D., Lucke, A.J., Skinner-Adams, T., Sahama, I., Haque, A., Do, T.A., McFadden, G.I., Fairlie, D.P., and Andrews, K.T. (2012). Antimalarial activity of the anticancer histone deacetylase inhibitor SB939. Antimicrobial agents and chemotherapy *56*, 3849-3856.

Surolia, N., and Padmanaban, G. (1992). de novo biosynthesis of heme offers a new chemotherapeutic target in the human malarial parasite. Biochemical and biophysical research communications *187*, 744-750.

Taylor, C.J., McRobert, L., and Baker, D.A. (2008). Disruption of a Plasmodium falciparum cyclic nucleotide phosphodiesterase gene causes aberrant gametogenesis. Molecular microbiology *69*, 110-118.

Thornalley, P.J., Strath, M., and Wilson, R.J. (1994). Antimalarial activity in vitro of the glyoxalase I inhibitor diester, S-p-bromobenzylglutathione diethyl ester. Biochemical pharmacology *47*, 418-420.

Triglia, T., Menting, J.G., Wilson, C., and Cowman, A.F. (1997). Mutations in dihydropteroate synthase are responsible for sulfone and sulfonamide resistance in Plasmodium falciparum. Proceedings of the National Academy of Sciences of the United States of America *94*, 13944-13949.

Tyler, P.C., Taylor, E.A., Frohlich, R.F., and Schramm, V.L. (2007). Synthesis of 5'-methylthio coformycins: specific inhibitors for malarial adenosine deaminase. Journal of the American Chemical Society *129*, 6872-6879.

Urscher, M., More, S.S., Alisch, R., Vince, R., and Deponte, M. (2012). Tight-binding inhibitors efficiently inactivate both reaction centers of monomeric Plasmodium falciparum glyoxalase 1. The FEBS journal *279*, 2568-2578.

Uyemura, S.A., Luo, S., Vieira, M., Moreno, S.N., and Docampo, R. (2004). Oxidative phosphorylation and rotenone-insensitive malate- and NADH-quinone oxidoreductases in Plasmodium yoelii yoelii mitochondria in situ. The Journal of biological chemistry *279*, 385-393.

Varadharajan, S., Dhanasekaran, S., Bonday, Z.Q., Rangarajan, P.N., and Padmanaban, G. (2002). Involvement of delta-aminolaevulinate synthase encoded by the parasite gene in de novo haem synthesis by Plasmodium falciparum. The Biochemical journal *367*, 321-327.

Vaughan, A.M., O'Neill, M.T., Tarun, A.S., Camargo, N., Phuong, T.M., Aly, A.S., Cowman, A.F., and Kappe, S.H. (2009). Type II fatty acid synthesis is essential only for malaria parasite late liver stage development. Cellular microbiology *11*, 506-520.

Vinayak, S., and Sharma, Y.D. (2007). Inhibition of Plasmodium falciparum ispH (lytB) gene expression by hammerhead ribozyme. Oligonucleotides *17*, 189-200.

Vivas, L., Easton, A., Kendrick, H., Cameron, A., Lavandera, J.L., Barros, D., de las Heras, F.G., Brady, R.L., and Croft, S.L. (2005). Plasmodium falciparum: stage specific effects of a selective inhibitor of lactate dehydrogenase. Experimental parasitology *111*, 105-114.

Waller, R.F., Keeling, P.J., Donald, R.G., Striepen, B., Handman, E., Lang-Unnasch, N., Cowman, A.F., Besra, G.S., Roos, D.S., and McFadden, G.I. (1998). Nuclear-encoded proteins target to the plastid in Toxoplasma gondii and Plasmodium falciparum. Proceedings of the National Academy of Sciences of the United States of America *95*, 12352-12357.

Waller, R.F., Ralph, S.A., Reed, M.B., Su, V., Douglas, J.D., Minnikin, D.E., Cowman, A.F., Besra, G.S., and McFadden, G.I. (2003). A type II pathway for fatty acid biosynthesis presents drug targets in Plasmodium falciparum. Antimicrobial agents and chemotherapy *47*, 297-301.

Wang, P., Wang, Q., Yang, Y., Coward, J.K., Nzila, A., Sims, P.F., and Hyde, J.E. (2010). Characterisation of the bifunctional dihydrofolate synthase-folylpolyglutamate synthase from Plasmodium falciparum; a potential novel target for antimalarial antifolate inhibition. Molecular and biochemical parasitology *172*, 41-51.

Wanidworanun, C., Nagel, R.L., and Shear, H.L. (1999). Antisense oligonucleotides targeting malarial aldolase inhibit the asexual erythrocytic stages of Plasmodium falciparum. Molecular and biochemical parasitology *102*, 91-101.

Webster, H.K., and Whaun, J.M. (1982). Antimalarial properties of bredinin. Prediction based on identification of differences in human host-parasite purine metabolism. The Journal of clinical investigation *70*, 461-469.

Witola, W.H., El Bissati, K., Pessi, G., Xie, C., Roepe, P.D., and Mamoun, C.B. (2008). Disruption of the Plasmodium falciparum PfPMT gene results in a complete loss of phosphatidylcholine biosynthesis via the serine-decarboxylase-phosphoethanolamine-methyltransferase pathway and severe growth and survival defects. The Journal of biological chemistry *283*, 27636-27643.

Wright, P.S., Byers, T.L., Cross-Doersen, D.E., McCann, P.P., and Bitonti, A.J. (1991). Irreversible inhibition of S-adenosylmethionine decarboxylase in Plasmodium falciparum-infected erythrocytes: growth inhibition in vitro. Biochemical pharmacology *41*, 1713-1718.

Yu, M., Kumar, T.R., Nkrumah, L.J., Coppi, A., Retzlaff, S., Li, C.D., Kelly, B.J., Moura, P.A., Lakshmanan, V., Freundlich, J.S., et al. (2008). The fatty acid biosynthesis enzyme FabI plays a key role in the development of liver-stage malarial parasites. Cell host & microbe *4*, 567-578.

Zhang, Y., and Meshnick, S.R. (1991). Inhibition of Plasmodium falciparum dihydropteroate synthetase and growth in vitro by sulfa drugs. Antimicrobial agents and chemotherapy *35*, 267-271.

Zhang, Y.A., Hempelmann, E., and Schirmer, R.H. (1988). Glutathione reductase inhibitors as potential antimalarial drugs. Effects of nitrosoureas on Plasmodium falciparum in vitro. Biochemical pharmacology *37*, 855-860.
